# Nonadherence to changing guidelines in early breast cancer over two decades

**DOI:** 10.1186/s12885-026-15731-x

**Published:** 2026-02-16

**Authors:** Annikki Aromaa-Häyhä, Päivi Auvinen, Nea Malila, Vesa Kataja

**Affiliations:** 1 Cancer Center, Kuopio University Hospital, Wellbeing services county of North Savo, P.O. Box 100, Kuopio, 70029 Finland; 2 Institute of Clinical Medicine, University of Eastern Finland, Kuopio, P.O. Box 1627, 70211 Finland; 3 Finnish Cancer Registry, Cancer Society of Finland, Mäkelänkatu 2, Helsinki, 00500 Finland; 4 South Savo Wellbeing Services County, Oncology Services Unit, Mikkeli Central Hospital, Porrassalmenkatu 35-37, Mikkeli, 50100 Finland

**Keywords:** Early breast cancer, Adjuvant systemic treatment, Guideline adherence, Overtreatment, Undertreatment

## Abstract

**Purpose:**

The incidence of breast cancer has increased, and many more cases are being detected with favorable tumor characteristics and in older age. The guidelines for treating early breast cancer have evolved as new treatments have become available. Our aim was to study the extent to which the guidelines reflect the real-world setting and whether guideline non-adherence is more frequent in some patient groups.

**Methods:**

Data from 803 women aged 40–84 years diagnosed with early breast cancer between 1992 and 2011 were retrieved from the clinical patient files and the Finnish Cancer Registry. To study adherence to existing national guidelines, undertreatment and overtreatment with respect to the guidelines were considered non-adherence, and adherence status was analyzed using multivariable regression models.

**Results:**

During the study period, the proportion of patients with guideline-based indications for systemic adjuvant treatment increased from 43.8% to 98.9%, while adherence to the guidelines decreased from 82.2% to 70.1%. Undertreatment was more frequent in the later years of the study (odds ratio [OR] 2.77; confidence interval [CI] 95% 1.67–4.59) compared to the beginning of the study, and more frequent among patients 70 years or older (OR 1.86; CI 95% 1.22–2.84) compared to patients 50–69 years of age. Undertreatment was more common in node-negative (T2-4N0) cases than in node-positive cases (OR 2.14; CI 95% 1.16–3.96).

**Conclusion:**

As treatment indications expand, it should be acknowledged that the results of clinical trials can only be applied to a portion of real-world patients, resulting in a decrease in guideline adherence. This dynamic should be considered in future guidelines and clinical trials.

**Supplementary Information:**

The online version contains supplementary material available at 10.1186/s12885-026-15731-x.

## Introduction

In recent decades, the incidence of breast cancer has increased, and among Finnish women, the lifetime risk of developing breast cancer was 13.3% in 2021 [1]. The introduction of population-based screening programs in the mid-1980s has increased the detection rate of breast cancer, especially that of early (i.e., nonmetastatic) breast cancer with favorable tumor characteristics [2]. In addition, improved imaging methods, a Western lifestyle, and aging, together with increasing general cancer awareness, have contributed to this development [3–5].

The guidelines for treating early breast cancer have evolved as new treatment options have become available. In Finland, the first national guideline for breast cancer diagnosis and treatment was published in 1994 [6]. Since then, the recommendations have been updated according to data from published studies and according to international guidelines. Nowadays, both the international and the Finnish national guidelines recommend the use of systemic treatment, at least endocrine treatment, for nearly all early breast cancer patients: only patients with small (< 0.5 mm) triple-negative breast cancer (TNBC) or human epidermal growth factor receptor 2 (HER2)-positive tumors may be considered eligible for undergoing no systemic therapy. In recent years, a shift has occurred from the adjuvant treatment setting to the neoadjuvant treatment setting, especially among patients with aggressive subtypes, such as TNBC and HER2-positive breast cancer [7]. However, considering patients with luminal breast cancer with a good prognosis, the expanding guidelines may cause concern regarding increased treatment intensity when more and more patients are exposed to treatment-related adverse effects.

With aging populations, more breast cancers are diagnosed at an older age [8]. Approximately one-third of real-world patients are older than the majority of patients in the clinical trials upon which the guidelines are based [9]. In addition, many real-world patients have comorbidities or reduced performance status that exclude them from clinical trials. This raises the question of how closely the guidelines can be followed in the real-world clinical setting and how vulnerable patients will be considered in future clinical trials, guidelines, and daily practice.

Given this context, this study explored how evolving breast cancer treatment methods have been implemented in the treatment of early breast cancer in the beginning of the 21st century. The particular aim of this study was to investigate the extent to which the guidelines reflect the real-world setting and whether non-adherence is more frequent in some patient groups.

## Patients and methods

The study population included 803 women aged 40–84 years diagnosed with early (nonmetastatic) breast cancer in the Kuopio University Hospital (KUH) catchment area in Eastern Finland. We compared the implementation of systemic treatment with endocrine treatment (ET) and chemotherapy (CT) in four time periods and three age groups, analyzing non-adherence to the existing national guidelines over a 20-year period from 1992 to 2011. Permission for this study was provided by the Finnish National Institute for Health and Welfare (THL/457/5.05.00/2012) and the Ethics Committee of the University of Eastern Finland.

Data were collected from the KUH clinical patient files (data on staging and treatment) and the Finnish Cancer Registry (patient identification). Individual data were linked with patients’ personal identification codes, which are unique for every permanent citizen in Finland. The data retrieval process has been detailed previously [10]. In all, 139 cases were excluded from the analysis due to in situ carcinomas and cases with metastatic or unknown metastatic status. This left 803 cases available for analysis. In addition, 17 patients who did not undergo radical surgery were included in adherence evaluations but excluded when studying use of adjuvant treatment.

To study the changes in different ages, the cases were divided into three age groups by the patients’ age at diagnosis: 40–49 years, 50–69 years, and 70 years or older (70+). During the study, population-based screening programs were organized by law in every municipality in Finland. Women aged 50–69 years were invited for screening biannually. However, in the first years of the study, the upper age limit for screening varied, ranging between 59 and 69 years in different municipalities. To study the change of treatment strategies over the period from 1992 to 2011, the data were divided into four equal five-year time periods: 1992–1996, 1997–2001, 2002–2006, and 2007–2011. From here forward, these are identified as the 1st, 2nd, 3rd, and 4th periods, respectively.

TNM classification of the tumors was based on an AJCC staging manual that was up to date relative to the year of diagnosis. Five different editions (3rd − 7th ) were used during the study period [11]. Estrogen receptor (ER) and progesterone receptor (PgR) status was determined by immunohistochemistry throughout the study period. The cutoff point for ER positivity was 10% [12]. Grading of the tumors was based on the Elston-Ellis system. The expression of HER2 amplification was determined by chromogenic in situ hybridization, and cancers with six or more gene copies were considered HER2-positive (HER2+).

During the study period, five national guidelines (1994, 1996, 1997, 1999, and 2007) were applied (Table 1). For the years 1992–1993, the 1994 recommendation was applied since that recommendation was largely based on the 1992 St. Gallen recommendation, which was used in practice. Between 1999 and 2007, no national recommendations were published, but the international guidelines were followed according to the consensus by the national breast cancer experts in the Finnish Breast Cancer Group. The guideline was considered to have taken effect from the beginning of the subsequent year of publication.

**Table 1:** Summary of adjuvant systemic treatment (AST) recommendations in National guidelines in Finland 1994–2007

year of guideline update	AST recommended	No AST	Recommended systemic treatment
1994	• pN1-3	• pT1N0	• Premenopausal : CMF x6
	• pT3-4	• pT2N0 without risk factors	• Postmenopausal : CMFx6 or AE for 3 years
	• pT2N0 and 2–3 risk factors: >2 cm, gr III, PgR negative		
1996	• pN1-3	• pT1N0	• Premenopausal : CMF x6
	• pT3-4	• pT2N0 without risk factors	• Postmenopausal : CMFx6 or AE for 3 years
	• pT2N0 and 2–3 risk factors: >2 cm, gr III, PgR negative		
1997	• pN1-3	• pT1N0	• Premenopausal : CMF x6
	• pT3-4	• pT2N0 without risk factors	• Postmenopausal : CMF x6 or AE for **3–5** years
	• pT2 and 2–3 risk factors: >2 cm, gr III, PgR negative		
1999	• pN1-3	**• pT1-2N0 and ER/PgR positive and gr I-II**	• Premenopausal : CMF x6 **OR CEF x6 if N2-3**
	• pT3-4		• **Postmenopausal: CT and/or AE for 3–5 years**
	**• T1 (> 1 cm)-pT2 and gr III**		
	**• T1 (> 1 cm)-pT2 and ER/PgR negative**		
2007	• pN1-3	**pT1N0 and**	**Low risk** ^**a**^ **:**
	**• pT2-4**	• **HER2-negative and**	• ET for ER/PgR positive*
			**intermediate risk** ^**b**^ **:**
	• **pT1N0 and**		•**ER/PgR positive: ET or ET + chemo**^**#**^
	•**ER/PgR positive or**	• **> 35 years and **	• **HER2+ (> 2 cm): trastuzumab + chemo (ET if ER/PgR positive)**
	•**<35 years or**	•** Gr 1**	• **TNBC: chemo**
	•**gr II-III**		**high risk** ^**c**^ **:**
			•** ER/PgR positive: chemo + ET**
			•** HER2+: chemo + trastuzumab (ET if ER/PgR positive)**
			• **TNBC: chemo**

*CMF* cyclofosfamide+metotrexare+fluorouracil, *AE* antiestrogen, *ER* estrogen receptor, *PgR* progesterone receptor, *gr*  gradus, *ET* endocrine treatment, *CEF* cyclofosfamide+epirubicin+fluorouracil, *TNBC* triple negative breast cancer

^a^Low risk: N0 and T1 and gr I and HER2- and no vascular invasion and age > 35 y

^b^intermediate: N1 (1–3 nodes) and ER/PgR + and HER2- OR N0 and T2-4 or grII-III or vascular invasion or ER/PgR – or HER2 + or < 35y

^c^high: N1 and ER/PgR- or HER2 + OR N2-3 (more than 4 nodes)

*premenopausal: AE, LHRH-analoque among young high risk patients, postmenopausal: aromatase inhibitors (AI) or AE

^#^ chemo = CEF/CMF or taxanes in combination with CMF or CEF in N + or HER2 + patients

The new features of the updated guidelines are bolded

To study the use of CT, four categories were formed: (1) CMF (cyclophosphamide, methotrexate, fluorouracil); (2) anthracycline-based regimens (epirubicin, doxorubicin, or mitoxantrone included in the CT combination); (3) taxane-based regimens; and (4) other (vinorelbine, cisplatin, mitoxantrone). HER2 + patients were also evaluated as to whether they were being treated with trastuzumab or not. The indications for adjuvant trastuzumab were a tumor >2 cm or node positivity since 2005. To study the use of ET, three categories were applied: (1) antiestrogens (AEs), either tamoxifen or toremifene; (2) aromatase inhibitors (AIs), either letrozole or anastrozole; and (3) therapeutic castration, either oophorectomy or luteinizing hormone-releasing hormone analogue therapy. (Radiotherapy of the ovaries was not used in this study.) Indication for ET was ER and/or PgR positivity.

To study adherence to the treatment recommendations, we evaluated whether cases had an indication for adjuvant treatment according to the existing recommendation and whether they were treated accordingly. Adherence was determined per each guideline at an individual patient level. Based on this, the cases were divided into three groups: adherent, undertreated, or overtreated. Cases in the guideline adherent group were either given the recommended adjuvant treatment or were not treated if there was no indication. Cases in the non-adherent group were either undertreated or overtreated. Undertreatment was defined as not receiving the recommended treatment. Any deviation from the systemic treatment recommendation (ET, CT, or both) was considered undertreatment. Cases in the overtreatment group were given treatment against the recommendation. Also, cases treated with both CT and ET when only one of them was indicated were considered overtreated.

For the literature review, a PubMed search was performed. Studies that were relevant to the study period and design were chosen.

Baseline characteristics were expressed as frequency counts and proportions, and the chi-square test was used for comparison. A Poisson regression model was used to assess guideline adherence, overtreatment, and undertreatment. Three regression analyses were performed: (1) adherent group (dependent variable) vs. undertreated + overtreated; (2) undertreated (dependent variable) vs. adherent group + overtreated; and (3) overtreated (dependent variable) vs. adherent group + undertreated. The analyses were adjusted for time period, age group, tumor grade, TNM class (N+, T2-4N0, and T1N0), and endocrine status (ER- and PgR-negative vs. ER- and/or PgR-positive or unknown). For some cases, endocrine status or grade was not available. The cases with unknown ER and PgR status were combined with ER- and PgR-positive cases for the analyses. The cases with unknown grade were considered as one subgroup of tumor grade. Four cases were missing TNM classification, and they were excluded from those analyses for which that information was needed.

The 4th period was separately analyzed to add HER2 status and triple negativity (i.e., ER, PgR, and HER2 negativity) as an adjustment to the multivariable model. If HER2 status was unknown, the cases were combined with the HER2-negative cases. Statistical analyses were performed using IBM SPSS version 26 for Windows.

## Results

### Description of the study population

The patient demographics in the four time periods are presented in Table 2. The majority of patients were 50–69 years old (52.1%), and the proportion of patients in this age group increased over time, from 48.8% in the 1st period to 60.1% in the 4th period. By contrast, the proportion of patients aged 40–49 years decreased from 23.8% to 10.1% from the 1st to the 4th time period. The largest proportion of patients had T1N0 cancer (43.3%), and the proportion was highest in the 4th period (48.3%). The proportion of node-positive patients was 41.0% overall, but decreased from 48.6% in the 3rd period to 37.6% in the 4th period. The proportion of ER- and PgR-negative cancer decreased from 20.6% in the 1st period to 11.8% in the 4th period.

**Table 2:** Descriptive statistics of patient demographics in the four time periods

	1992–1996 *n* = 214*	1997–2001 *n* = 238#	2002–2006 *n* = 173	2007–2011 *n* = 178	All *n* = 803
	*n* (%)	*n* (%)	*n* (%)	*n* (%)	*n* (%)
Age group					
40–49	51 (23.8)	54 (22.7)	28 (16.2)	18 (10.1)	151 (18.8)
50–69	104 (48.6)	113 (47.5)	94 (54.3)	107 (60.1)	418 (52.1)
70+	59 (27.6)	71 (29.8)	51 (29.5)	53 (29.8)	234 (29.1)
TNM classification					
N+	78 (37.0)	100 (42.2)	84 (48.6)	67 (37.6)	329 (41.0)
T2-4N0	32 (15.2)	45 (19.0)	20 (11.6)	25 (14.0)	122 (15.2)
T1N0	101 (47.9)	92 (38.8)	69 (39.9)	86 (48.3)	348 (43.3)
Grade					
Gr I–II	134 (62.6)	156 (65.6)	109 (63.0)	111 (62.4)	510 (63.5)
Gr III	49 (22.9)	46 (19.3)	48 (27.7)	52 (29.2)	195 (24.3)
Gr unknown	31 (14.5)	36 (15.1)	16 (9.2)	15 (8.4)	98 (12.2)
Endocrine status					
ER- and PgR-	44 (20.6)	37 (15.5)	27 (15.6)	21 (11.8)	129 (16.1)
ER/PgR+	110 (51.4)	174 (73.1)	140 (80.9)	149 (83.7)	573 (71.4)
Unknown	60 (28.0)	27 (11.3)	6 (3.5)	8 (4.5)	101 (12.6)
HER2 status					
HER2+	-	-	33 (19.1)	33 (18.5)	66 (8.2)
HER2-/unknown	-	-	140 (80.9)	145 (81.5)	737 (91.8)
TNBC	-	-	5 (2.9)	13 (7.3)	18 (2.2)

*N +*  node positive, *Gr*  grade, *ER* estrogen receptor, *PgR* progesterone receptor, *HER2*  human epidermal growth factor 2, *TNBC* triple-negative breast cancer (i.e., ER-, PgR-, and HER2-)

* three cases were missing TNM classification and were thus excluded from the analysis of TNM classification

# one case was missing TNM classification and was thus excluded from the analysis of TNM classification

### Implementation of adjuvant systemic therapy

Adjuvant CT was given to approximately one-third (35.4%) of the 786 patients who were operated on with curative intention. There was a significant increase in the proportion of patients treated with adjuvant CT: 19.3%, 27.1%, 48.0%, and 52.5% in the 1st, 2nd, 3rd, and 4th periods, respectively (*p* < 0.001). Among patients treated with adjuvant CT, the proportion of patients treated with CMF significantly decreased (*p* < 0.001), while the use of anthracyclines and taxanes increased in the 3rd and 4th periods (*p* < 0.001) (Table 3).

**Table 3:** Descriptive statistics of adjuvant systemic treatment (AST) after radical surgery in different time periods#

	1992–1996 *n* = 202	1997–2001 *n* = 236	2002–2006 *n* = 171	2007–2011 *n* = 177	All *n* = 786	*p*
	*n* (%)	*n* (%)	*n* (%)	*n* (%)	*n* (%)	
AST	85 (42.1)	115 (48.7)	115 (67.3)	133 (75.1)	448 (60.0)	< 0.001
CT	39 (19.3)	64 (27.1)	82 (48.0)	93 (52.5)	278 (35.4)	< 0.001
CMF^a^	24 (61.5)	44 (68.8)	26 (31.7)	23 (24.7)	117 (42.0)	< 0.001
anthracycline^a^	18 (46.2)	22 (34.4)	57 (69.5)	63 (67.7)	160 (57.6)	< 0.001
taxane^a^	1 (2.6)	1(1.6)	27 (32.9)	43 (46.2)	72 (25.9)	< 0.001
anthracycline + taxane^a^	1 (2.6)	1 (1.6)	24 (29.3)	37 (39.8)	63 (22.7)	< 0.001
other^a^	0	0	1 (1.2)	1 (1.1)	2 (0.7)	ns
HR positive/unknown	158 (78.2)	199 (84.3)	144 (84.2)	157 (88.7)	658 (83.7)	ns
ET^b^	42 (26.6)	71 (35.7)	92 (63.9)	117 (74.5)	322 (48.9)	< 0.001
antiestrogen (AE)^c^	42 (100)	70 (98.6)	78 (84.8)	70 (59.8)	260 (39.5)	< 0.001
aromatase inhibitor (AI)^c^	1 (2.4)	7 (9.9)	26 (28.3)	60 (51.3)	94 (14.3)	< 0.001
AE + AI^c^	1 (2.4)	6 (8.5)	12 (13.0)	13 (11.1)	32(4.7)	ns
castration^c^	0	1 (1.4)	3 (3.3)	5 (4.3)	9 (1.4)	ns

# a single patient may have had more than one type of treatment, making it possible for the sum of one column to exceed 100%

^a^ proportions calculated among patients treated with chemotherapy (CT)

^b^ proportions calculated among hormone receptor (HR) positive/unknown patients

^c^ proportions calculated among patients treated with endocrine therapy (ET)

There were 658 ER- and/or PgR-positive or unknown cases, of whom adjuvant ET was given to 322 patients (48.9%). The proportion of receptor-positive/unknown patients treated with adjuvant ET increased over time: 26.6%, 35.7%, 63.9%, and 74.5% in the 1st, 2nd, 3rd, and 4th periods, respectively (*p* < 0.001). Among the 322 patients treated with adjuvant ET, 80.7% were treated with AEs. The proportion of patients treated with AEs decreased over time (*p* < 0.001), while the proportion of patients treated with AIs or both AEs and AIs increased (*p* < 0.001) (Table 3).

Trastuzumab was not available during the 1st and 2nd periods. In the 3rd period, 27.3% (9 out of 33) of the HER2 + patients were treated with trastuzumab—two of them within the FinHER-trial [13] from 2002 to 2003 and seven after the publication of the first positive results of adjuvant trastuzumab in 2005 [14]. In the 4th period, 66.7% (22 out of 33) of HER2 + cases received trastuzumab as adjuvant treatment: 11 out of 15 HER2+/N+ patients and 11 out of 18 HER2+/N0 patients.

### Time trends in adjuvant systemic treatment indications and guideline adherence

There was a time-related increase in the proportion of patients with a guideline-based indication for systemic treatment, from 43.8% in the 1st period to 98.9% in the 4th period. However, the proportion of all patients treated according to the recommendations decreased over time: 82.2%, 88.2%, 79.8%, and 70.1% in the 1st, 2nd, 3rd, and 4th periods, respectively (*p* < 0.001) (Table 4).

**Table 4:** Descriptive statistics of guideline (GL) adherence for adjuvant systemic treatment (AST) in different time periods

	1992–1996 *n* = 214	1997–2001 *n* = 238	2002–2006 *n* = 173	2007–2011 *n* = 177^a^	All *n* = 802
	*n* (%)	*n* (%)	*n* (%)	*n* (%)	*n* (%)
Adherence	176 (82.2)	210 (88.2)	138 (79.8)	124 (70.1)	648 (80.8)
AST according to GL	71 (33.2)	107 (45.0)	93 (53.8)	122 (68.9)	393 (49.0)
No AST according to GL	105 (49.1)	103 (43.3)	45 (26.0)	2 (1.1)	255 (31.8)
Non-adherence	38 (17.6)	28 (11.8)	35 (20.2)	53 (29.9)	154 (19.2)
Undertreatment	26 (12.1)	19 (8.0)	14 (8.1)	50 (28.2)	109 (13.6)
Overtreatment	12 (5.6)	9 (3.8)	21 (12.1)	3 (1.7)	45 (5.6)

^a^ one patient was excluded due to unknown adherence status

Time-related changes in guideline adherence show decreasing adherence and an increasing proportion of undertreatment, indicating that almost one-third of the patients were not treated according to guidelines in the last time period.

**Fig. 1:**
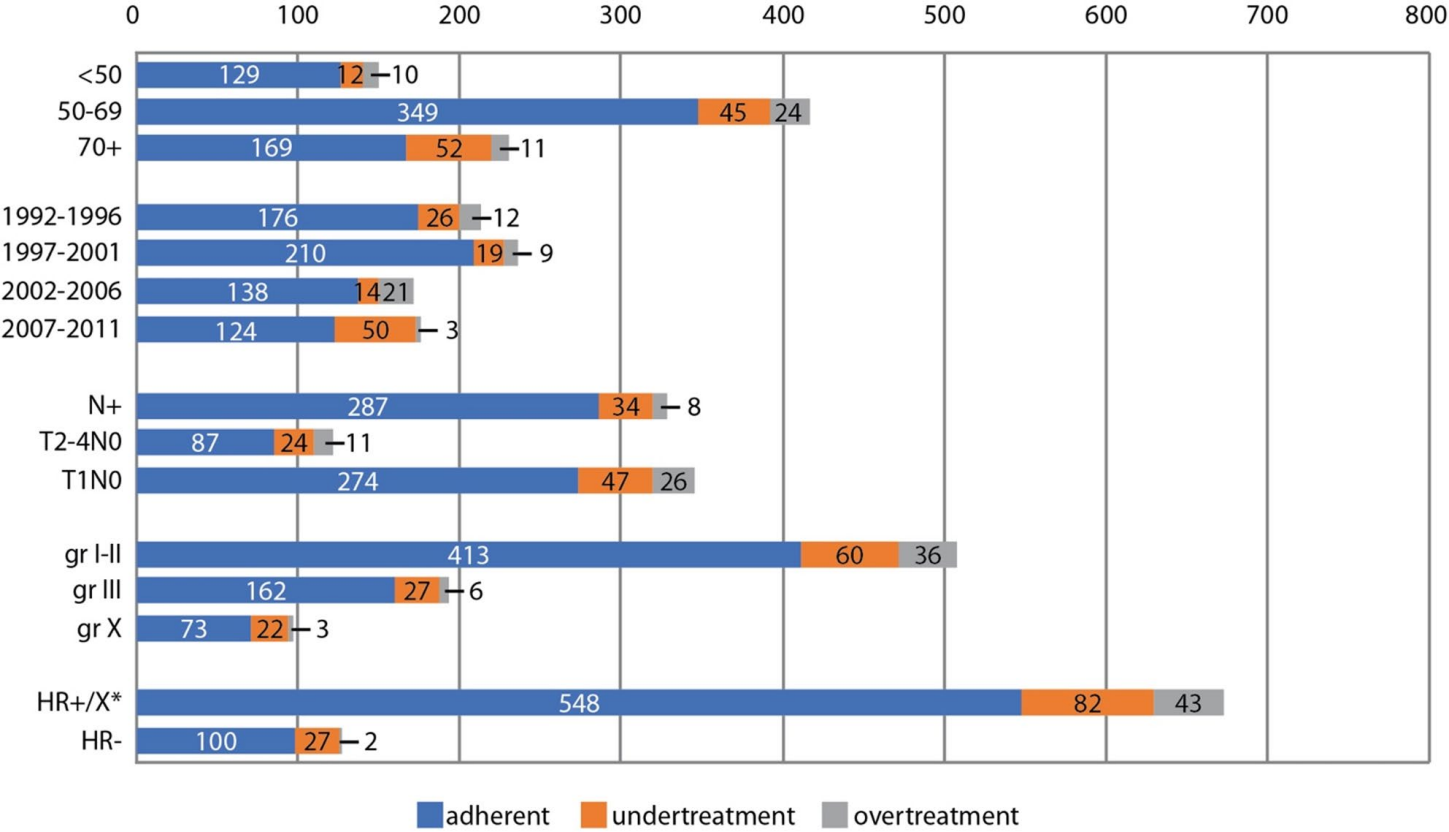
The number of patients in the guideline adherence, undertreatment, and overtreatment groups among different patient subgroups. gr = grade, X = unknown, HR = hormone receptor (estrogen and/or progesterone receptor)

The data regarding guideline adherence, undertreatment, and overtreatment in different patient subgroups are presented in Fig. 1.

### Undertreatment

Undertreatment (*n* = 109) was statistically significantly more frequent among patients 70+ (odds ratio [OR] 1.86; confidence interval [CI] 95% 1.22–2.84) compared to women aged 50–69 years, but not compared to patients aged 40–49 years. Undertreatment was more frequent in the 4th period than the 1st period (OR 2.77; CI 95% 1.67–4.59), but there was no statistical difference in the 2nd and 3rd period compared to the 1st. Compared to the node-positive cases, undertreatment was more frequent among node-negative cases, especially among T2-4N0 cases (OR 2.14; CI 95% 1.16–3.96). Also, undertreatment was more frequent among ER- and PgR-negative patients than among ER- and/or PgR-positive patients or patients with unknown ER and PgR status (OR 2.32; CI 95% 1.42–3.80) (Table 5).

**Table 5:** Multivariable Poisson regression analysis of guideline adherent treatment, undertreatment and overtreatment stratified for categorial variables

Categorial predictors	Guideline adherent treatment (*n* = 648)^a^			Undertreatment (*n* = 105)^b^			Overteatment (*n* = 45)^c^			All (*n* = 798)*
	*n*	OR	CI95%	*n*	OR	CI95%	*n*	OR	CI95%	*n*
Age group										
40–49	129	0.98	0.80–1.21	12	0.90	0.47–1.72	10	1.32	0.63–2.80	151
50–69	49	1.0		45	1.0		24	1.0		418
70+	169	0.88	0.73–1.07	52	1.86	1.22–2.84	11	0.81	0.39–1.70	232
Time period										
1992–1996	176	1.0		26	1.0		12	1.0		214
1997–2001	210	1.06	0.87–1.29	19	0.68	0.37–1.27	9	0.66	0.28–1.57	238
2002–2006	138	0.93	0.74–1.17	14	0.78	0.38–1.52	21	2.37	1.16–4.86	173
2007–2011	124	0.82	0.65–1.04	50	2.77	1.67–4.59	3	0.29	0.08–1.05	177
TNM classification										
T1N0	274	0.90	0.76–1.07	47	1.30	0.83–2–04	26	2.93	1.31–6.58	347
T2-4N0	87	0.83	0.65–1.06	24	1.73	1.01–2.95	11	4.47	1.78–11.38	122
N+	287	1.0		34	1.0		8	1.0		329
Hormone Receptor status										
Er/PgR + or unknown	548	1.0		82	1.0		43	1.0		673
ER- and PgR-	100	0.89	0.71–1.13	27	2.32	1.42–3.76	2	0.26	0.06–1.19	129
Grade										
gr I-II	413	1.0		60	1.0		36	1.0		509
gr III	162	1.06	0.87–1.30	27	0.83	0.49–1.39	6	0.64	0.26–1.57	195
gr unknown	73	0.93	0.73–1.20	22	1.72	1.03–2.87	3	0.47	0.14–1.54	98

*N +*  node positive, *Er* estrogen receptor, *PgR* progesterone receptor, *gr * grade, *HER2*  human epithelial growth factor 2

*Four cases were excluded from analysis due to missing TNM-status and one due to unknown adherence status

Table of three Poisson regression analysis combined: (a) adherent group (dependent variable) vs. undertreated + overtreated, (b) undertreated (dependent variable) vs. adherent group + overtreated and (c) overtreated (dependent variable) vs. adherent group+undertreated

### Overtreatment

Overtreatment (*n* = 45) was less frequent than undertreatment (Table 4). In the multivariable analysis, cases in the 3rd period were more likely to be overtreated compared to those in the 1st period (OR 2.37; CI 95% 1.16–4.86), but not in the 2nd and 4th period. Overtreatment was more frequent in node-negative cases than in the node-positive cases of T2-4N0 (OR 4.47; CI 95% 1.78–11.38) and T1N0 (OR 2.93; CI 95% 1.31–6.58). Overtreatment was not associated with age group, endocrine status, or grade (Table 5).

### The 4th period

In the multivariable analysis of the 4th period (*n* = 177), only TNM classification was significantly associated with guideline adherence. Compared to node-positive patients, T1N0 patients were more often undertreated (OR 3.48 CI 95% 1.60–7.54), but no difference was detected in patients with T2-4N0. There were 32 HER2 + cases available for analysis; 13 out of 15 HER2+/N+ patients and 13 out of 17 HER2+/N0 patients were treated in accordance with the guidelines. HER2 status was not associated with undertreatment or overtreatment.

In the 4th period, there were 13 TNBC cases, 10 of which were treated according to the guidelines. TNBC status was not associated with undertreatment or overtreatment. Multivariable Poisson regression analysis of guideline adherent treatment, undertreatment and overtreatment stratified for categorial variables in the 4th period is shown in Additional file 1.

## Discussion

In this study of 803 early breast cancer cases from 1992 to 2011, we found that the proportion of patients with guideline-based indications for systemic adjuvant treatment increased from 43.8% to 98.9%. At the same time, adherence to existing guidelines decreased from 82.2% to 70.1%. Undertreatment with respect to guidelines was more common than overtreatment. Undertreatment was almost threefold in the 4th period compared to the 1st period and almost twofold among older (70+) cases compared to the 50–69 age group.

The proportion of patients treated with adjuvant CT increased from 19.2% in the 1st period to 52.2% in the 4th period, and patients treated with adjuvant ET from 26.6% to 74.5%, respectively. During the 1st and 2nd periods, either CT or hormonal therapy was recommended as a systemic therapy. In the latest guidelines in the study period, ET was recommended for almost all ER- or PgR-positive cases, which means that very few patients with small ER- and PgR-negative tumors were excluded from systemic adjuvant treatment recommendations. Thus, during the 4th period only a very small proportion of patients (1.1%) had no guideline-based indication for systemic adjuvant treatment. Despite the increase in the number of patients receiving systemic treatment, the change was not as substantial as the expansion of the systemic treatment indications in the recommendations. Therefore, undertreatment with respect to the existing guidelines increased almost threefold from the first to the last period. Similarly, a Dutch registry study between 1990 and 2012 [15] found that expanding recommendations resulted in an increased chance of undertreatment during the most recent years of the study period, with 10% of the patients being undertreated for ET and 29% being undertreated for CT. Clinical guidelines are intended to standardize treatment practices and promote evidence-based decision-making. However, many of the patients treated in daily practice do not fit into the inclusion and/or exclusion criteria of the studies behind the formation of the guidelines.

In the present study, women 70 + were more likely to be undertreated than other age groups. The influence of age was also seen in two Dutch registry studies. A study by Verschoor et al. [15] showed that being 70 years or older was a risk factor for undertreatment. In another Dutch registry study [16], guideline adherence was significantly lower among women aged 80 years or older. In Finland, 28% of new breast cancer patients between 2007 and 2011 were 70 years or older at the time of diagnosis, while between 2017 and 2021, that figure had risen to 37% [1]. Similarly, in many Western countries the population is aging rapidly, with approximately one-third of breast cancers occurring in women over 70 years of age [9]. By contrast, in the trials on which the guidelines are based, the proportion of older patients is very low. The recommendations have been extrapolated from the results of younger and otherwise fit women to older age groups and otherwise more fragile populations. In addition to age, comorbidities are another reason for guideline non-adherence [17]. In our catchment area of KUH, the overall morbidity is the highest in Finland [18]. The incidence of obesity-related health issues, such as cardiovascular diseases and type 2 diabetes, is especially high. Consequently, these factors may reflect the undertreatment of breast cancer patients in our daily practice.

The guideline adherence for systemic therapy was highest in the 2nd period (1997–2001), when nearly 90% of the patients were treated according to the recommendations. During the first two periods of the study (1992–2001), the guidelines were simple: they recommended either CT or ET, mainly for high-risk patients, especially node-positive cases. In addition, the changes in guidelines were minor between the 1st and 2nd period. In the 3rd period, novel treatments (taxanes and AIs, expansion of adjuvant indication to smaller tumors) were adopted in clinical practice before respective amendments were made to the national guidelines, which could explain why overtreatment increased almost threefold in that period. Unfavorable clinicopathological characteristics have been shown to decrease the chance of undertreatment [15]. Similarly, in the present study, undertreatment was less frequent in node-positive cases than in node-negative cases. Node positivity has been an indication for adjuvant treatment since the first national guidelines in 1994 and is thus well established.

This study investigated the evolving systemic treatments of early breast cancer that were adopted in clinical practice at the beginning of the 21st century. During the study period, the guideline-based indications for systemic adjuvant treatment have come to include almost all breast cancer patients. Our results show that adherence to existing guidelines was highest among high-risk patients, while undertreatment was more frequent among older patients. In clinical trials, guidelines are based upon the efficacy of a treatment that was usually evaluated among women much younger and healthier than many of the patients in daily clinical practice. In addition to efficacy, the significance of effectiveness—that is, treatment efficacy in real-life patients—is highlighted in the European Society of Medical Oncology (ESMO) Clinical Practice Guidelines [19]. Systematic assessment of quality-of-life factors and treatment-related toxicity (i.e., effectiveness) has been shown to improve overall survival and to decrease the risk of mortality among cancer patients [20, 21].

There were some limitations in this retrospective study. Data on grade and endocrine receptor status were not available for all the patients in the first years of the study. The missing information may have increased the use of CT, since PgR negativity was considered one of the high-risk factors among T3-4N0 patients in the national guidelines published in 1994 and 1996. However, during the first two periods, both CT and ET were allowed as forms of adjuvant therapy, which means that the missing data most likely did not affect the adherence status. Another limitation was that systematic HER2 testing was not available until 2005; hence, the association of biological subtype to adherence status was studied only in the last period. Also, we did not have information on comorbidities or performance status to evaluate the association of these factors with non-adherence.

To conclude, the development of systemic treatments in early breast cancer has been extensive. However, the results of clinical trials can only be applied to a portion of real-world patients, resulting in a practically unavoidable decrease in guideline adherence. This discrepancy should be acknowledged and accepted in clinical decision-making, but also considered in future guidelines and clinical trials.

## Supplementary Information

Supplementary Material 1

## Data Availability

The datasets generated and/or analyzed during the current study are not publicly available due to fact that they contain information that could compromise research participant privacy but may be available from the corresponding author on reasonable request with required permissions.

## Abbreviations

TNBC: triple-negative breast cancer
HER2: human epidermal growth factor receptor 2
KUH: Kuopio University Hospital
ET: endocrine treatment
CT: chemotherapy
ER: estrogen receptor
PgR: progesterone receptor
HER2+: human epidermal growth factor receptor 2 positive
CMF: cyclophosphamide, methotrexate, fluorouracil
AEs: antiestrogens
AIs: aromatase inhibitors
Gr: grade
GL: guideline
AST: adjuvant systemic treatment
ESMO: European Society of Medical Oncology
