# Supplementary material for: Nonadherence to changing guidelines in early breast cancer over two decades
Source: BMC Cancer. 2026 Feb 16;26:530. doi: 10.1186/s12885-026-15731-x (PMC13123143; doi:10.1186/s12885-026-15731-x)
Supplement: Supplementary file 1 — Supplementary Material 1 [file 12885_2026_15731_MOESM1_ESM.docx]

**Additional file 1.** Multivariable Poisson regression analysis of guideline adherent treatment, undertreatment and overtreatment stratified for categorial variables in the 4^th^ period (2007 – 2011)

| Categorial predictors | Guideline adherent treatment (n=124)^a^ | | | Undertreatment (n=50)^b^ | | | Overteatment (n=3)^c^ | | | All (n=177) |
| --- | --- | --- | --- | --- | --- | --- | --- | --- | --- | --- |
|  | n | OR | CI95% | n | OR | CI95% | n | OR | CI95% | n |
| Age group |  |  |  |  |  |  |  |  |  |  |
| 40-49 | 15 | 1.09 | 0.62 – 1.91 | 2 | 0.56 | 0.13 – 2.42 | 1 | NR |  | 18 |
| 50-69 | 79 | 1.0 |  | 26 | 1.0 |  | 0 | NR |  | 105 |
| 70+ | 30 | 0.77 | 0.50 – 1.16 | 22 | 1.80 | 1.00 – 3-25 | 2 | NR |  | 54 |
|  |  |  |  |  |  |  |  |  |  |  |
| TNM classification |  |  |  |  |  |  |  |  |  |  |
| T1N0 | 45 | 0.63 | 0.42 – 0.93 | 38 | 3.48 | 1.60 – 7.54 | 2 | NR |  | 85 |
| T2-4N0 | 21 | 1.04 | 0.61 – 1.75 | 4 | 1.09 | 0.32 – 3.75 | 0 | NR |  | 25 |
| N+ | 58 | 1.0 |  | 8 | 1.0 |  | 1 | NR |  | 67 |
| Hormone Receptor status |  |  |  |  |  |  |  |  |  |  |
| Er/PgR/HER2+ | 114 | 1.0 |  | 47 | 1.0 |  | 3 | NR |  | 164 |
| TNBC | 10 | 0.96 | 0.44 – 2.07 | 3 | 0.87 | 0.18 – 4.16 | 0 | NR |  | 13 |
|  |  |  |  |  |  |  |  |  |  |  |
| Grade |  |  |  |  |  |  |  |  |  |  |
| gr I-II | 74 | 1.0 |  | 9 | 1.0 |  | 3 | NR |  | 111 |
| gr III | 44 | 1.12 | 0.71 – 1.77 | 33 | 0.71 | 0.24 – 2.47 | 0 | NR |  | 52 |
| gr unknown | 6 | 0.64 | 0.28 – 1.50 | 8 | 1.89 | 0.90 – 3.98 | 0 | NR |  | 14 |
|  |  |  |  |  |  |  |  |  |  |  |
| HER2 status |  |  |  |  |  |  |  |  |  |  |
| HER2- | 98 | 1.0 |  | 44 | 1.0 |  | 3 | NR |  | 145 |
| HER2+ | 26 | 0.98 | 0.61 – 1.57 | 6 | 1.06 | 0.41 – 2.77 | 0 | NR |  | 32 |

N+= node positive, Er= estrogen receptor, PgR= progesterone receptor, gr = grade, HER2 = human epithelial growth factor 2

Table of three Poisson regression analysis combined: a) adherent group (dependent variable) vs. undertreated + overtreated, b) undertreated (dependent variable) vs. adherent group + overtreated and c) overtreated (dependent variable) vs. adherent group+undertreated
